# Maladie de Basedow révélant une tuberculose thyroïdienne

**DOI:** 10.11604/pamj.2017.26.163.11562

**Published:** 2017-03-21

**Authors:** Hicham Attifi, Abdelhamid Messary

**Affiliations:** 1 Service d’Otorhinolaryngologie et Chirurgie Cervico-faciale, Hôpital Militaire Moulay Ismaïl, Meknès, Maroc

**Keywords:** Glande thyroïde, tuberculose, maladie de Basedow, Thyroid gland, tuberculosis, Graves’ disease

## Image en médecine

La tuberculose thyroïdienne est rare même dans les pays endémiques tel le Maroc et dont le diagnostic peut poser problème en absence d’éléments d’orientation cliniques ou biologiques. Elle est exceptionnellement révélée par une maladie de Basedow. Patiente âgée de 48 ans, suivie au service d’endocrinologie pour maladie de Basedow, ayant reçu un traitement à base d’antithyroïdiens de synthèse et adressée au service d’otorhinolaryngologie pour rechute à l’arrêt du traitement en vue d’une cure chirurgicale. L’examen physique a objectivé une thyroïde augmentée de taille avec présence d’un nodule basilobaire gauche ferme, d’un diamètre de 2 cm. La naso-fibroscopie a montré des cordes vocales mobiles. Les aires ganglionnaires étaient libres. L'échographie cervicale a objectivé des nodules hypoéchogènes et hyperéchogènes au dépend des 2 lobes. Le bilan hormonal a révélé un profil d'une hyperthyroïdie périphérique avec une TSH effondrée. La radiographie thoracique ne montrait pas de lésions médiastino-pulmonaires. Plusieurs pathologies peuvent s'associer exceptionnellement à une maladie de Basedow tel qu'un lymphome, une tuberculose ou un carcinome. La patiente a bénéficié d'une thyroïdectomie totale. L'examen anatomopathologique a objectivé un parenchyme thyroïdien hyperplasique avec présence en plusieurs endroits de granulomes épithélioïdes et giganto-cellulaires avec des foyers de nécrose caséeuse. Le diagnostic de tuberculose thyroïdienne a été posé. Elle n'y avait pas d'autres localisations tuberculeuses. La patiente a reçu un traitement antituberculeux pour une durée totale de 12 mois associé à une hormonothérapie substitutive à vie. L'évolution était favorable sans notion de récidive. Le recul était de 2 ans.

**Figure 1:**
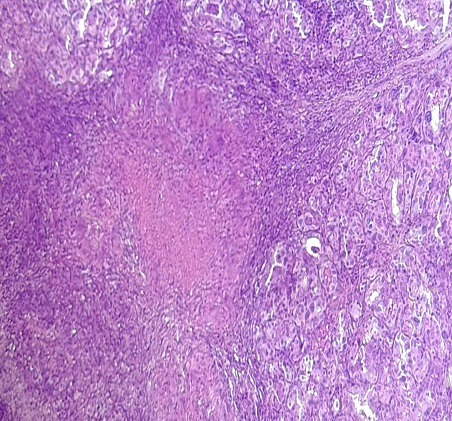
Parenchyme thyroïdien caractérisé par une nette hyperplasie avec accentuation de la lobulation évoquant une maladie de Basedow et siège de granulome épithéloïde et gigantocellulaire avec foyers de nécrose caséeuse (HE x 200)

